# One Abutment One Time: A Multicenter, Prospective, Controlled, Randomized Study

**DOI:** 10.3390/ijerph17249453

**Published:** 2020-12-17

**Authors:** José Vicente Ríos-Santos, Gregorio Tello-González, Pedro Lázaro-Calvo, Francisco Javier Gil Mur, Blanca Ríos-Carrasco, Ana Fernández-Palacín, Mariano Herrero-Climent

**Affiliations:** 1 Advanced Periodontics, Facultad de Odontología, Universidad de Sevilla, C/Avicena S/N, 41009 Seville, Spain; gretello@hotmail.com (G.T.-G.); pedro@lazarocalvo.com (P.L.-C.); brios@us.es (B.R.-C.); 2 Technological Health Research Center, Biomaterials of the Faculties of Medicine and Dentistry, International University of Cataluña, 08034 Barcelona, Spain; xavier.gil@uic.cat; 3 Department of Biostatistic, Dental School, Universidad de Sevilla, 41009 Sevilla, Spain; afp@us.es; 4 Porto Dental Institute, 4150-518 Porto, Portugal; dr.herrero@herrerocliment.com

**Keywords:** connection and disconnection abutment, implant-abutment connections, implant bone loss, dental implants, implant collar, implant neck, marginal bone level

## Abstract

*Aim*: (PRIMARY) Assess the changes in bone level (6 and 12 months after implant placement) between the test (definitive abutment (DEF)) and control (healing abutment (HEA)) groups. (SECONDARY) Assess the changes in bone level (6 and 12 months after implant placement) between the 1 mm high abutment group and 2 mm abutment group. Evaluate changes in implant stability recorded with analysis of the resonance frequency (RFA) Osstell system, at 6 and 12 months after implant placement, between the control group (HEA) and test (DEF). For the DEF group, the abutment was placed at the time of the surgery and was never removed. For the HEA group, the abutment was removed three times during the manufacture of the crowns. The abutments used were 1 mm high (Subgroup A) and 2 mm high (Subgroup B). Materials and methods: A total of 147 patients were selected between 54.82 ± 11.92 years old. After implant placement, patients were randomly distributed in the DEF and HEA group. After the implant placement, a periapical radiograph was taken to assess the peri-implant bone level; the same procedure was carried out 6 and 12 months post-placement. To compare the qualitative variables between the groups (HEA/DEF), the Chi-square test was used; for quantitative (MANOVA). *Results*: After a year, the accumulated bone loss was 0.48 ± 0.71 mm for the HEA group and 0.36 ± 0.79 mm for the DEF group, without statistical significance. Differences were only found due to timing (time) between 0 and 6 months (=0.001) and 0 and 12 months (0.001), with no differences attributable to the study groups (DEF and HEA). The accumulated bone loss (1 year) was 0.45 ± 0.78 mm for the 1 mm abutment group and 0.41 ± 0.70 mm for the 2 mm abutment group (*p* = 0.02). No differences were observed in implant stability between groups. *Conclusions*: The “One Abutment—One Time” concept does not reduce peri-implant bone loss compared to the connection–disconnection technique. The height of the abutment does influence bone loss: the higher the abutment, the lower the bone loss.

## 1. Introduction

In the last decade, research on implants has been focused on investigating the factors that maintain peri-implant tissues in a healthy and stable long-term situation [1,2,3,4].

One of the factors studied was the platform-switching concept for bone level implants. Platform switching is done when an abutment of less diameter than an implant is connected, moving away from the abutment–implant interface. Furthermore, the biological width is made horizontally versus the original one, which is vertical [1]. Another advantage of platform switching is that the presence of an inflammatory infiltrate is far away from the interface. In addition, the stress concentration at the bone crest moves on to the axial axis of the implant [2,3].

Concerning the biological width [4], the effect on the peri-implant tissues of the connection and disconnection of the healing abutments or screws during the prosthesis fabrication procedure has to be investigated. Furthermore, the level of fiber insertion appears to be fractured and therefore bacterial penetration is likely, due to the lack of epithelial sealing. The biological width is located in the contaminated area [5], although, the bone was reabsorbed by the inflammatory infiltrate produced, after which, the biological space is located apically from the starting point because space was created for the epithelium and connective tissue between the bone crest and the breach or contaminated area [6].

The placement of the definitive prosthetic abutment at the time of implant placement surgery, without being removed, a concept known as “One Abutment—One Time” [7] seems to favor the maintenance of the peri-implant bone level and therefore of the soft tissues.

In 1997, Abrahamsson [8] published a histological study containing a test group, wherein he connected and disconnected the healing screw once every 6 months and a control group in which he kept the abutment intact. The results obtained were statistically significant greater bone loss and recession of the peri-implant mucosa on the test group subjects versus the control group ones. Moreover, this same protocol has been studied in both animals (Becker et al. [9], Rodriguez et al. [10], Alves et al. [11]) and humans (Canullo et al. [7], Degidi et al. [12], Grandi et al. [13], Koutouzis et al. [14], Grandi et al. [15], Degidi et al. [16], Luongo et al. [17], Molina et al. [18], Praça et al. [19]) where the results show less peri-implant bone loss by the “One Abutment—One Time” protocol patients, although this slight difference may be associated with the small sample size of different studies or the different variables involved such as the moment of implant placement or the restorative protocol in each study.

Another factor to take into account is the thickness of the peri-implant mucosa—significantly greater bone loss has been observed in cases of tissues with a thickness lower than 2 mm [20,21]. Subsequently, the relationship of mucosa thickness with implants rehabilitated with platform change, or without platform change, was analyzed without finding any differences between the groups [22], giving even greater importance to the thickness of the tissue in terms of bone loss. 

The correct vertical insertion of the implant at the bone crest is an important aspect of implants with an internal connection [23,24]. In implants placed subcrestal, the bone loss has been lower with a platform switching design, finding statistical significance among those placed at the crestal level associated with 1 mm abutments between 2 mm abutments placed 3 mm subcrestal [25]. 

Based on these findings, we concluded that bone loss might be favored by the establishment of the biological width and the presence of inflammatory infiltrate around the crown–implant gap, so the use of taller abutments can reduce peri-implant bone loss [25,26,27], by moving away from the gap and thus, the contaminated area of the bone crest [5].

Our study assessed the application of the concept “One abutment—One time” (no disconnection of the abutment) in implants at the bone level with a change of platform in the maintenance of the peri-implant crestal bone in the face of repeated disconnection of the abutment of healing in the same type of implants. If we do not find differences between placing the abutment on the day of surgery or later, the selection of the abutment and its height could be deferred to the moment when the peri-implant tissues are completely healed after surgery, probably with a better clinical result.

### Objectives

1. Assess the changes in bone level (6 and 12 months after implant placement) between the test (definitive abutment (DEF)) and control (healing abutment (HEA)) groups.
2. Assess the changes in bone level (6 and 12 months after implant placement) between the 1 mm high abutment group and 2 mm abutment group.
3. Evaluate changes in implant stability recorded with analysis of the resonance frequency (RFA) Osstell system, at 6 and 12 months after implant placement, between the control group (HEA) and test (DEF).

## 2. Materials and Methods

### 2.1. Study Design

This study was a randomized, double blind, multicenter clinical study (20 centers), in which all surgeons were specialists in implantology and had more than 10 years of experience in implant surgery.

Patients were divided into two groups:

(A) Control (healing abutment—HEA-) in which the healing abutment was removed three times during the procedure.
(B) Test (definitive abutment—DEF-) whose definitive abutment was placed surgery day and was never removed.

The prosthetic abutments used were 1 or 2 mm for both the DEF group (surgery day placement) and the HEA group (placed the prosthesis day).

The centers were located in Spain and Portugal, monitored by the University of Seville (Spain). The study was approved by the Ethics Committee of the University of Seville in March 2016 (Nº: 0962-N-15) following the Declaration of Helsinki (1964) and its amendments (Tokyo, 1975; Venice, 1983; Hong Kong, 1989; Somerset, 1996; Edinburgh, 2000).

### 2.2. Study Population

The patient recruitment period was from April 2016 to September 2016.

Criteria of exclusion and inclusion were as follows:

Patients over 18 years of age were included, with dental absence/s in FDI positions no: 4-5-6-7 both maxillary and mandibular, with a healing period post-extraction of more than 4 months, with vertical and horizontal bone availability that allowed the placement of Klockner VEGA implants^®^ 4.0(Klockner Implant System, SOADCO S.L., Andorra) or 4.5 mm of diameter and 8, 10, 12 or 14 mm length; patients without signs of periodontal disease activity according to Matchei’s criteria [28]: the absence of suppuration, plaque index, bleeding less than 15% [29], and compromised to attend their reviews during the study. In the horizontal direction, the existence of a minimum bone tissue of 2 mm around the implant contour was considered necessary. In the vertical sense, the existence of sufficient bone height would be necessary for the placement of the implant of the desired length, with a safety margin of at least 2 mm in the case of implants placed above the inferior dental nerve and that did not require the elevation of the maxillary sinus floor to increase bone availability. No patient was excluded due to their smoking habit.

HIV+ patients, bruxists, or with diseases that affect the immune system and/or bone metabolism who were either previously or currently in treatment with bisphosphonates, or who had undergone bone regeneration treatments in the area to be treated in the previous 6 months were excluded, as well as patients who at the time of implant placement required bone regeneration of more than 25% of the implant surface or presented active, recurrent, or unresolved infection in the implant placement area or adjacent to the location implant. 

### 2.3. Sample Size

Using the nQuery Advisor program, and taking as a reference the results of the radiographic follow-up of similar studies (Canullo [4]) a significance level of 0.05 was determined, with a power of 80% estimating a sample size of N = 70 patients per group.

### 2.4. Implants and Attachments

VEGA Surface ContacTi^®^ implants were used (Klockner Implant System, SOADCO S.L., Andorra), with a bone level type and platform switching. A conical implant with an internal connection was to be placed at the crestal level with the bioactive surface, 4.0 or 4.5 mm diameter with platform change of 0.35 and 0.60 mm, respectively, and 8, 10, 12, and 14 mm in length were placed. The ContacTi surface is a bioactive subtraction surface with thermochemical treatment and a medium roughness Sa 1.6 μ [29].

**Figure 1:**
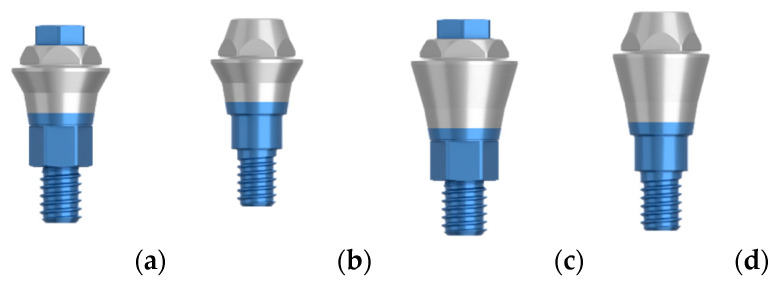
(**a**) Permanent 1 mm unit; (**b**) permanent 1 mm multiple; (**c**) permanent 2 mm unit; (**d**) Permanent 2 mm multiple.

The healing abutment used (HEA group) was 5 mm high and conical in shape. The definitive abutments, placed on the day of surgery in the DEF group and on the day of the prosthesis installation in the HEA group, were Permanent^®^ abutments (Klockner Implant System, SOADCO, Andorra), 1 or 2 mm high for single or multiple screw-retained restorations (depending on the thickness of the mucosa)—see Figure 1.

### 2.5. Study Development

#### 2.5.1. Surgical Procedure

All patients were treated with antibiotics (amoxicillin 750 mg/8 h; 7 days or clindamycin 300 mg/8 h; 7 days in allergic patients) and anti-inflammatory medications (ibuprofen 600 mg/8 h; 4 days) together with gastric protector (omeprazole 20 mg/24 h; 7 days). Said treatment began 30 min before the start of the surgical procedure. The surgeries were performed under local anesthesia with articaine 40 mg/mL + epinephrine 0.01 mg/mL (Artinibsa^®^, Inibsa Dental, Barcelona, Spain), the flap was raised to full-thickness performing crestal incision preserving 2 mm of keratinized mucosa both in buccal and lingual/palatal areas and after exposing the bone crest, the drilling sequence and insertion procedure proposed by the manufacturer (SOADCO) of Klockner VEGA implants were followed. The most coronal part of the implants was placed 1 mm subcrestal in the most apical area of the bone crest; at the mesial-distal level, they were placed at least 1.5 mm to the adjacent tooth and at least 3 mm in case if there is an adjacent implant. The implant diameter was selected to maintain a minimum of 2 mm of bone tissue around the full diameter of the implant. After insertion of the implant, the bone quality was recorded according to the Lekholm and Zarb classification [30] (I, II, III, or IV).

#### 2.5.2. Randomization

After insertion of the implant, each patient was assigned to the corresponding group in a random (balanced) manner by opening sealed envelopes sent directly with each implant. All implants of a patient (whether single or multiple) were assigned to either:

- Control group: healing abutment (HEA Group): connection–disconnection of the healing abutment.
- Test group: definitive abutment: (DEF group): placed on the day of surgery following the concept of ‘One Abutment—One Time’.

#### 2.5.3. Implant Stability

After insertion of the implant, the RFA values (analysis of the resonance frequency) were recorded by placing the Smart-Pegs^®^ (Type 26) for Klockner Vega^®^ implants (Osstell^®^, Gothenburg, Sweden) at 5 Ncm. RFA measurements were obtained via ISQ (Implant Stability Quotient) values from vestibular and mesio-distally using the Osstell ISQ^®^ device (Osstell^®^, Gothenburg, Sweden), recording the mean of the two records obtained as the value. If the stability recorded by RFA did not reach the value of 60 ISQ points, the implant was excluded from the study.

In the following reviews, the RFA measurements were recorded with Smart-Pegs^®^ (Type 72) on the permanent abutment.

The decision to place 1 or 2 mm permanent abutments^®^ was determined by the clinician after assessing the thickness of the mucosa and selecting the one considered appropriate in each particular case. The abutment (DEF group) was placed on the day of surgery with the same insertion torque (10 Ncm) as the healing abutment (HEA group). The final torque (25 Ncm) of the permanent abutment^®^ on the day of prosthesis placement was established for both groups.

Supramid^®^ coaxial synthetic suture^®^ (Aragó^®^, Barcelona, Spain) 5/0 or similar was used as a suture, obtaining primary closure. Postoperative instructions were given, and the patients continued with the medical regimen established and described previously.

If at the time of implant insertion, there was no adequate bone availability, clinically determined primary stability was not achieved, or bone regeneration procedures required was more than 25% of the implant surface, the patient was withdrawn from the study without considering it either as a treatment or an implant failure.

#### 2.5.4. Prosthetic Procedure

For the patients included in the HEA group, the decision to place permanent abutments^®^ 1 or 2 mm was taken by the clinician the day of taking impressions, which were taken 12 weeks after surgery using screw-retained impression copings for the Klockner VEGA system^®^ with perforated trays, using medium viscosity polyethers as an impression material (Impregum 3M-ESPE^®^, St. Paul, MN, USA). Moreover, abutments were torqued (25 Ncm).

On one side, in the HEA group, the impression was taken by connecting the impression copings directly to the implant, removing the healing screw for the first time; in the DEF group, the impression was taken by connecting the impression copings to the permanent abutment^®^. On the other side, in the HEA group, the selection of the height of the prosthesis abutment was made on the master cast. Firstly, the prostheses were constructed following conventional crown and bridge manufacturing standards. Secondly, at 14 weeks, the metallic structure was tested. Thirdly, in the HEA group, the healing screw was removed a second time and was repositioned until the prosthesis was placed, whereas, in the DEF group, the permanent abutment^®^ was never removed. Finally, at 16 weeks, the prosthesis was placed, which was performed according to the proposed screw-retained prosthesis protocol with permanent^®^ abutments by the manufacturer of the Klockner^®^ implant system applying 15 Ncm of torque to the screws. In both groups, the prosthesis was placed on an abutment and not directly to the implant, in such a way, that the examiners could not differentiate by radiographs which group each patient belonged to and thus making sure that the simple-blind of the examiners marking and measuring the radiographs was maintained. X-rays on the day of surgery were taken in both groups with the healing abutment; once the radiography was performed, the permanent abutment was placed in the DEF group. The examiner who analyzed the measurements never knew which group he belonged to.

#### 2.5.5. Clinical Evaluation

The peri-implant tissue health was monitored clinically during the follow-up 6 and 12 months visits. Before starting the study, all the patients underwent scale-out and oral hygiene training. In addition, the parameters modified plaque index (Mombelli [31]) and modified bleeding index (Mombelli [31]) on mesial, distal, vestibular, and lingual or palatal surfaces were registered by a periodontal probe (PCP UNC-15). After that, implant stability was recorded with the RFA Osstell system^®^. Finally, the ISQ value was measured to the Permanent abutment^®^ in the DEF group and directly to the implant in the HEA group.

#### 2.5.6. Radiographic Analysis

**Figure 2:**
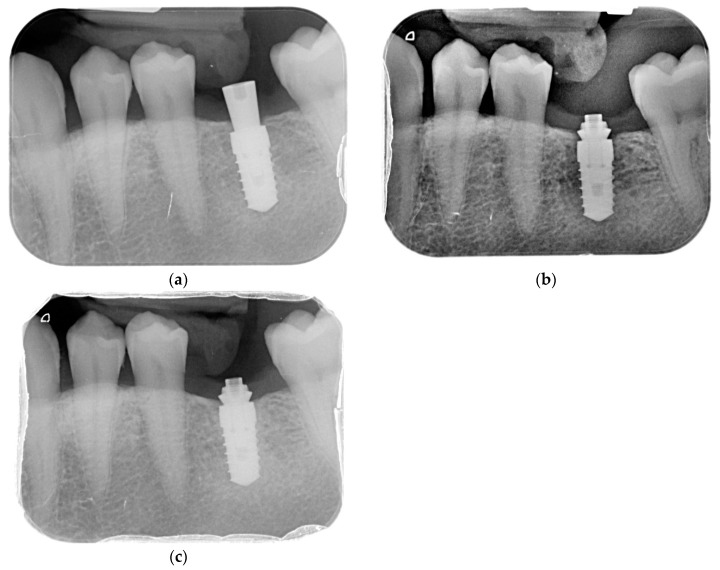
Case evolution. (**a**) Rx Baseline; (**b**) Rx 6 M; (**c**) Rx 12 M.

To evaluate interproximal bone levels, (individualized bite holder) standardized intra-oral radiographs were performed using the long-cone paralleling technique. The radiographs were taken before implant placement, postoperatively, at 2, 6, and 12 months—see Figure 2.

**Figure 3:**
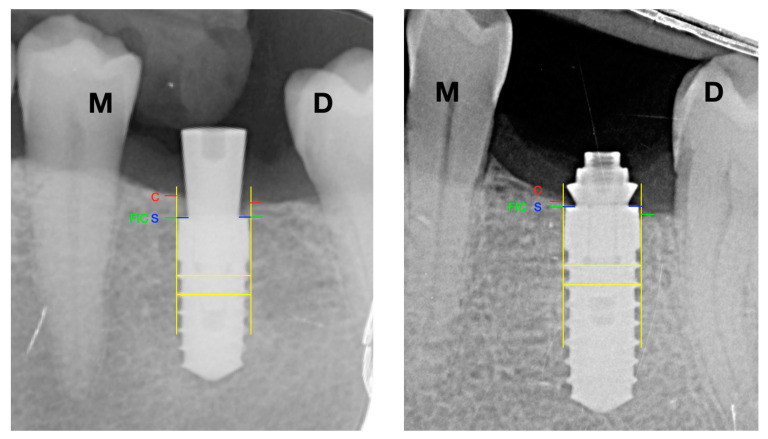
Radiographic analysis. S: implant shoulder; C: bone crest; FIC: first bone to implant contact; M: mesial; D: distal; SC distance: marginal bone level at the crest; S-FIC distance: marginal bone level at the implant.

Consequently, all radiographs were calibrated by assessing their possible distortion, using the distance between the threads of the implant body as a known value. All implants were evaluated by drawing vertical lines parallel to the long axis of the implant, taking the implant platform (S) as the starting point, then the mesial and distal crestal bone level of each implant was recorded. Besides cases of presenting bone tissue on the platform, the measurement was from platform to bone crest (C). In addition, in cases where the bone crest (C) was apical to the implant platform, the distance between the platform (S) and the first bone-implant contact (FIC) was measured—see Figure 3.

Negative values (−) were established in case the implant platform was subcrestal and positive values (+) if it was coronal to the bone crest.

For the statistical analysis, the value was obtained using a mean between the mesial and distal values of radiographic bone loss.

All the initial radiographs (baseline) were made with a healing screw (5 mm), in this way, the operator in charge of measuring the crestal bone level could not radiographically know which group each implant belonged to during the study.

Two operators, previously calibrated (BRC and GTG), marked the turns, implant platform (S), bone crest (C), and first bone-implant contact (FIC) and measured the distances between turns, SC, and S-FIC using the ImageJ software (National Institutes of Health, Madison, MD, USA).

The examiners were calibrated by evaluating 30 radiographs at three different times with an interval of three days [32], resulting in an intraclass correlation coefficient of 0.947.

### 2.6. Statistical Method

The quantitative variables were summarized with means and standard deviations or, if the distributions presented a marked asymmetry, with medians and percentiles (P25 and P75), whereas the qualitative variables were described with frequencies and percentages.

Intraclass correlation coefficients and their 95% confidence intervals were obtained between examiners. To compare the qualitative variables between the groups (HEA/DEF), the Chi-square test, the Chi-square test with continuity correction, or Fisher’s exact test (for sparsely populated 2 × 2 tables) were applied.

To study the follow-up of the quantitative variables (ISQ, Global bone, etc.) in the two groups (HEA/DEF), the multivariate procedure General Linear Model of Repeated Measures (MANOVA measures) with two samples was used. The intra-subject factor was the measurement times (6 and 12 months); the inter-subject factor was the group (HEA/DEF). Using this statistical analysis procedure, we compared the individual effects of the factors and the interactions between them. When the within-subject factor and/or the interaction between the two factors (intra and inter) were significant, Bonferroni multiple comparison tests were performed. Likewise, 95% confidence intervals were calculated, and profile graphs were drawn.

The null hypothesis is: there are changes in the maintenance of peri-implant bone levels when the definitive abutment is placed on the day of surgery or when the prosthesis is installed.

Data analysis was performed with the statistical package IBM SPSS Statistics 26.0 (IBM Corporation, Armonk, NY, USA) for Windows.

## 3. Results

During the recruitment period, 160 patients (252 implants) enrolled in the study, out of which: 13 patients abandoned the study, 8 did not attend the 6 month review, 4 did not attend the 12 month review, and 1 patient was discarded in the 6 month check-up due to becoming pregnant and being unable continue with the control check-ups.

During the study period, four failed implants corresponding to three centers were counted (one center had two failures), the four implants failed during the first two months post-surgery. Finally, the survival rate was 98.42%.

**Figure 4:**
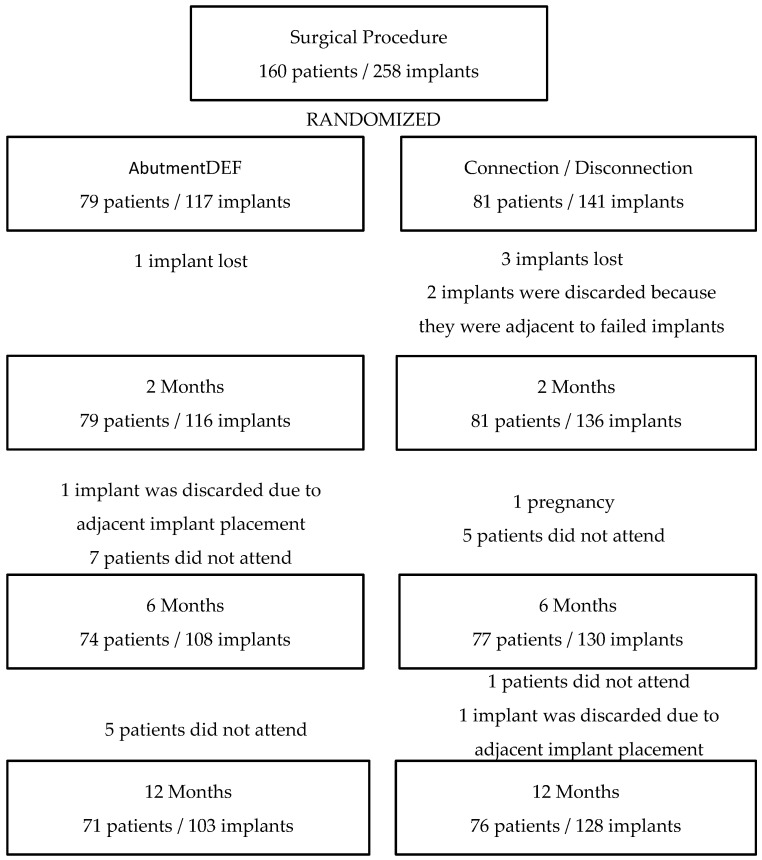
Population Diagram.

After that, four implants (four patients) dropped-out, two were adjacent to failed implants, one was the placement of an adjacent implant between 2 and 6 months of follow-up, and the last one was void because a tooth extraction was made and an adjacent implant was placed during the follow-up period, considering that the bone regularization can interfere with the relevant bone needed for the implants (Figure 4). 

To sum up, the sample size was 147 patients: 76 (51.7%) HEA group and 71 (48.3%) DEF group (without statistical significance) with a total of 231 implants (128 HEA group and 103 DEF group) (without statistical significance).

Not statistical significance were found in the 1 and 2 mm abutments groups. For the implants on a 1 mm abutment, 119 implants were rehabilitated, of which, 62 (48.43%) correspond to the control group (HEA) and 57 (55.34%) to the test group (DEF). For the implants on a 2 mm abutment, 112 implants were rehabilitated, of which, 66 belonged to the HEA group (51.57%) and 46 (44.66%) to the DEF group.

In this study, for the 147 patients enrolled in this study, 81 were women (55.1%) and 66 were men (44.9%). This population was distributed among the groups as follows: 35 (46.1%) men and 41 (53.9%) women were in the HEA group; 40 (56.3%) men and 31 (43.7%) women were in the DEF group, with a mean age of 54.89 years without statistical significance among the HEA group (55.80 ± 10.93) and DEF group (53.85 ± 12.91). 

With regard to smoking, each patient was previously registered as either a non-smoker (57.1%), ex-smoker (8.8%), <10 cigarettes (13.6%), and 10–20 cigarettes (1.4%), without statistical significance between HEA and DEF groups.

Concerning bone quality [30] recorded on the day of surgery, not statistical significance was observed between the HEA and DEF group; we found that the bone quality of the population was 13 (5.6%) type I bone, 127 (55%) type II bone, 88 (38.1%) type III, and 3 (1, 3%) type IV bone. 

Regarding the type of prosthesis, we found 133 (57.6%) single crowns and 98 (42.4%) multiple prostheses, without statistical significance was observed between the HEA and DEF group between HEA and DEF groups. 

A total 91.8% of admitted patients had no history of periodontal disease; 8.2% had been treated successfully. Furthermore, no statistical significance was found between the groups surrounding patients with periodontal disease; 5 (6.66%) in the HEA group and 7 (9.99%) in the DEF group presented signs of non-active periodontal disease (Table 1).

When evaluating the modified plaque index [31], statistical significance was not found between the HEA group and the DEF group at the 6 and 12 month check-ups in any locations (*p* > 0.005)—see Table 2 and Table 3. Not statistical significance was found concerning the modified bleeding index [31] at different locations studied at the 6 and 12 month check-ups (*p* > 0.005)—see Table 4 and Table 5.

The mean bone level (mesial–distal) peri-implant on the day of surgery was −0.70 ± 0.62 mm for the HEA group and −0.79 ± 0.80 mm for the DEF group. After 1 year, the peri-implant bone level was −0.21 ± 0.71 mm for the HEA group and −0.43 ± 0.79 mm for the DEF group. Statistical significance was not found between groups. Cumulative bone loss was 0.48 ± 0.71 mm for the HEA group and 0.36 ± 0.79 mm for the DEF group, with no statistical significance between the groups. Statistically significant differences were only found between the different study moments, between 0–6 months (HEA: 0.46 ± 0.66 mm; DEF: 0.40 ± 0.66 mm) and between 0–12 months (HEA: 0.48 ± 0.71 mm; DEF: 0.36 ± 0.79 mm), regardless of the group (*p* < 0.001). The data of the bone level relationship between test and control groups are shown in Table 6.

The mean peri-implant bone level on the day of surgery was −0.65 ± 0.63 mm for the 1 mm group and −0.84 ± 0.77 mm for the 2 mm abutment group. After 1 year, the peri-implant bone level was −0.20 ± 0.78 mm for the 1 mm group and −0.43 ± 0.70 mm for the 2 mm abutment group. The accumulated bone loss was 0.45 ± 0.78 mm for the 1 mm group and 0.41 ± 0.70 mm for the 2 mm abutment group, observing statistical significance (*p* < 0.002) between the 1 mm and 2 mm groups. The data on the bone level relationship between groups 1 and 2 mm are shown in Table 7.

The area of the result that showed significant differences (*p* < 0.002) was the height of the abutment and bone loss over time, regardless of the group in which it was placed—the higher the height of the abutment, the less bone loss.

For record-keeping, the mean RFA values were recorded (Table 8), the HEA group presented 77.18 ± 9.30 ISQ on the day of surgery; the DEF group presented 78.36 ± 8.13 ISQ. At 6 months, the HEA group had 79.67 ± 8.18 ISQ and the DEF group 76.54 ± 9.41 ISQ; at 12 months, the HEA group 80.07 ± 9.01 ISQ and DEF 76.51 ± 12, with 46 ISQ. Not statistical significance (*p* = 0.731) was found throughout the study period.

## 4. Discussion

### 4.1. Maintenance of the Preload According to the Type of Connection

In the present study, peri-implant bone loss was investigated by studying the implants previously described, which were placed in the healed bone and screwed prosthetic at 16 weeks. The patients were randomized after implant placement and after primary stability was measured to avoid bias on the part of the operators at the time of randomization. After this, a homogeneous population was observed with no differences between the groups in regard to age, gender, bone quality, smoking, implant stability, or abutment height (Table 1). No statistically significant differences were found between the placement of the definitive abutment on the day of surgery without being removed at any other time or following the concept “One Abutment—One Time”, compared to the conventional prosthetic procedure in which the healing screw is removed at least three times. The evolution of the peri-implant bone level is similar in both groups, presenting the greatest loss in the first 6 months, with bone loss being stable in the two groups between 6 and 12 months (Figure 1). Even without statistically significant differences, the tracking in peri-implant bone loss accumulated at the one-year follow-up in the DEF group (0.36 ± 0.79 mm) is more favorable than the one in the HEA group (0.48 ± 0.71 mm).

Statistical significance was not found between HEA and DEF groups regarding plaque control and bleeding at 6 and 12 months, therefore, the improved response by the DEF group is not due to the better or worse control of plaque by the patient.

When evaluating previous studies with similar characteristics (RCTs), we found a diversity of criteria such as the placement of immediate implants (Canullo [4]; Degidi [16]; Grandi [15]) or healed ridge (Grandi [13]; Koutouzis [14]; Molina [18]), or studies that placed both immediate implants and healed ridge (Luongo [17]), where we observed no difference between groups during the first 4 months—e.g., the period in which the connection—disconnection process of the healing screw was carried out—however, in the HEA group, there was a greater accumulated bone loss at the three-year mark (0.50 mm) compared to the DEF group (0.07), finding statistical significance (*p* = 0.007) [33]. We found no statistically significant differences between immediate implants associated with immediate loading without occlusion and those placed in a healed ridge, considering both options as treatment alternatives.

Focusing on studies where healed ridge implants were placed [13,14,18,19], in two of them, Koutouzis [14] and Praça [19] statistical significance (*p* > 0.05) were not found in peri-implant bone loss. First [14], they compared the HEA group (0.28 ± 0.16 mm) and the DEF group (0.13 ± 0.20 mm) at the 6 month follow-up (Praça [19]). At two years of evolution, they found no statistically significant differences (HEA: 0.81 ± 0.15 mm; DEF: 0.61 ± 0.10 mm). In addition, Praça [19] only found statistical significance between different moments of the study such as between 0 and 2 months (HEA: 0.36 ± 0.10 mm; DEF: 0.70 ± 0.12 mm) and between 2 and 6 months (HEA: 0.65 ± 0.14 mm; DEF: 0.11 ± 0.11 mm); however, in the other two studies, they did find statistical significance: Grandi [13] (*p* < 0.001) found a bone loss of 0.09 ± 0.03 mm in the DEF group and 0.44 ± 0.03 mm in the HEA group, whereas the Molina study [18](*p* = 0.028) found a bone loss of 0.59 ± 0.32 mm in the DEF group and 1.21 ± 0.82 mm in the HEA group. Furthermore, the HEA group presented greater bone loss than the DEF group in both studies. In our study, no statistical differences were found, just as in the aforementioned studies; however, bone loss was greater in the group in which the healing screw was removed and replaced up to three times, unlike in previous studies. In these studies, implants were placed at the crestal level, whereas in our study, implants were placed at the subcrestal level.

A significant factor to take into account is the position of the implant platform at the time of implant placement. In our case, the indication was to place the implant subcrestally due to the expected remodeling of the ridge [34,35], with the HEA group at 0.70 mm and the DEF group at 0.79 mm distance between the implant platform and the bone crest on the day of surgery. Clinically, they try to leave the implants 1 mm subcrestal, but the reality is that there are small variations between patients. Even more so considering that it is a multicenter study in which several surgeons participate. It is intended to reflect the reality of daily practice, which is not a mathematical science. On this value, there is also variability between the studies, in which implants were placed in the healed ridge (Grandi [13]; Koutouzis [14]; Molina [18]; Praça [19]) in all of them, the platform was left at the crestal level; however, in implants that were immediately placed (Canullo [7]; Degidi [16]; Grandi [15], Luongo [17]), the position of the platform was subcrestal. All studies were performed with internal connection implants and the presence of platform switching. 

Another cofactor that can affect bone remodeling and consequently the results of the study is the time of the prosthetic loading, both study groups underwent the prosthetic procedure at the same time, the only difference was that, during this period, in the HEA group the healing screw was removed to perform the impression taking (12 weeks) and subsequent tests until the placement of the definitive prosthesis (16 weeks). In this sense, all studies [12,13,14,18,19] performed the prosthetic protocol or placement-disconnection of the healing abutment at the same time.

In our study, as in previous studies [25,26,27], statistical significance (*p* = 0.002) has been found when comparing peri-implant bone loss in relation to the height of the intermediate abutments. Galindo [27], with a follow-up of 18 months, observed better results with regard to peri-implant bone loss in those implants that were placed with abutments of more than 2 mm in height compared to abutments of less than 2 mm (<2 mm: 0.049 ± 0.004; ≥2 mm: 0.024 ± 0.005) (*p* = 0.009). White [25] compared abutments of 1 vs. 3 mm, finding statistical significance both at 3 months (0.83 ± 0.19 mm vs. 0.14 ± 0.08 mm) and at 6 months of follow-up (0.91 ± 0.19 mm vs. 0.11 ± 0.09 mm). Novoa [26], after a 3-year follow-up comparing abutments of 1 vs. 2.5 mm in height, concluded that peri-implant bone loss is greater in those cases in which short abutments (<2 mm) are used, finding statistical significance at 12 (1 mm: 0.82 ± 0.10 mm; 2.5 mm: 0.2 ± 0.28 mm), 24 (1 mm: 1.27 ± 1.02 mm; 2.5 mm: 0.22 ± 0.37 mm) and 36 (1 mm: 1.23 ± 1.61 mm; 2.5 mm: 0.35 ± 0.62 mm) months of follow-up. 

Due to the fact that we have not found differences between placing the abutment on the day of surgery or later, the selection of the abutment and its height can be deferred to the moment when the peri-implant tissues are completely healed after surgery; independently of that we use a conventional workflow or CAD/CAM techniques for prosthetic rehabilitation [36].

The use of resonance frequency analysis (RFA) is the most reliable method to assess implant stability over time [37]. In our study, the values were recorded on the day of surgery, at 6 months, and at 12 months post-implant placement. On the day of surgery, all records (RFA) were taken with Smart-Pegs directly from the implant, whereas at the 6 and 12-month revisions, the Smart-Pegs were screwed to the prosthesis abutments. The values at 2 months of follow-up were not recorded since, at that time, it would have been necessary to measure the implant in the HEA group and the abutment in the DEF group since it was never removed, thus avoiding the existing differences in the values measured to implant or abutment [38]. The values were considered high on the day of surgery (HEA: 77 ± 9.30 ISQ; DEF: 78.36 ± 8.13 ISQ) being slightly higher in the DEF group, these values remained stable both in the 6 month review (HEA: 79.67 ± 8.18 ISQ; DEF: 76.54 ± 9.41 ISQ) and in the 12 month review (HEA: 80.07 ± 9.01 ISQ; DEF: 76.51 ± 12.46 ISQ). A slight decrease was observed in the DEF group after 6 months, a common trend after osseointegration when high primary stability values are obtained [39]. statistical significance (*p* = 0.731) was not found between groups (HEA/DEF) or between the different times of the study (surgery, 6 months, and 12 months) in each group. The results of our study are similar to those presented by Praça [19], a study with similar methodology comparing HEA and DEF groups, which also specifically presented similar results both on the day of surgery (HEA: 77.1 ± 5.5 ISQ; DEF: 75.1 ± 5.8 ISQ) and 6 month follow-up (HEA: 72.0 ± 3.2 ISQ; DEF: 74.3 ± 2.8 ISQ) without finding statistical significance concerning the RFA values between the groups, nor between the different moments of the study, presenting little variability between the day of surgery and 6 months.

In Cornelini’s study [40], whose objective was to evaluate the success and stability of implants placed in posterior mandibular sectors associated with immediate loading at 12 months, results were observed on the day of surgery (72 ± 5.7 ISQ) and their evolution up to 12 months (74.5 ± 7.3 ISQ) concluded with results similar to ours, although they are not comparable due to the methodological differences between both studies. In a previous study of ours [41], with screw-shaped, tissue level implants (Essential Cone, Klockner Implant System, SOADCO, Engordany, Andorra) with two different collar heights (0.7 mm or 1.5 mm), high ISQ values were also obtained, which we consider to be directly related to the success of dental implant treatments. The ISQ on the day of the surgery was 68.61 ± 10.35; at 8 weeks, it was 70.61 ± 7.5; and at 2 years, it was 74.39 ± 9.64.

### 4.2. Importance of This Study for the Clinic

The intention of the study is to clinically verify an existing trend, as suggested by some authors, which would be to place the abutment on the day of surgery in the case of using implants with the switching platform concept, in order to prevent possible crestal bone loss and possible influence on this bone remodeling of the height of the abutment used. If this situation were confirmed, the clinical attitude would be conditioned.

### 4.3. Limitation of this Study

For aesthetic reasons, the selection of the height of the abutment is not random and is left to the discretion of who selects it, and this may condition possible crestal bone loss. Another determining factor is the use of different implant diameters and therefore different platform change dimensions in combination with the different heights of the abutments.

## 5. Conclusions

We conclude that the “One Abutment—One Time” concept does not significantly reduce the peri-implant bone loss compared to the prosthetic protocol in which the healing screw is removed up to three times during the prosthesis procedure.

After the results obtained in our study, we can determine that the height of the prosthetic abutment has a statistically significant influence on bone loss, concluding that there is greater peri-implant bone loss using 1 mm high abutments compared to using 2 mm high abutments.

More studies are needed to determine if the “One Abutment—One Time” concept has a decisive influence on peri-implant bone loss since the different studies found in the literature have some limitations—that they share similar criteria.

## Figures and Tables

**Table 1:** Population.

	Test (DEF)	Control (CIC)	*p*-Values
GENDER			0.868
Male	40 (56.3%)	35 (46.1%)	
Female	31 (43.7%)	41 (53.9%)	
AGE	53.85 ± 12.91	55.80 ± 10.93	0.388
ISQ			0.058
0 M	78.36 ± 8136	77.18 ± 9309	
2 M	74.63 ± 8857	80.58 ± 5365	
6 M	76.54 ± 9410	79.67 ± 8188	
12 M	76.51 ± 12,463	80.07 ± 9.019	
SMOKER			0.299
Non smoker	55 (77.46%)	46 (60.52%)	
Former smoker	7 (9.86%)	15 (19.73%)	
<10 Cigarettes	9 (12.68%)	13 (17.11%)	
10–20 Cigarettes		2 (2.64%)	
BONE QUALITY			0.7
Type I	6 (5.8%)	7 (5.5%)	
Type II	62 (60.2%)	65 (50.8%)	
Type III	32 (31.1%)	56 (43.7%)	
Type IV	3 (2.9%)		
PERIODONTAL			0.554
Yes	7 (9.9%)	5 (6.66%)	
No	64 (90.1%)	71 (93.4%)	
PILLARS			0.632
1 mm	57 (55.34%)	62 (48.43%)	
2 mm	46 (44.66%)	66 (51.57%)	

**Table 2:** Modified Plaque Index, 6 months.

	Visible with Probe				Visible				Abundant			
	M	V	D	L/P	M	V	D	L/P	M	V	D	L/P
HEA	75.8%	77.3%	76.6%	76.6%	12.5%	10.9%	11.7%	10.2%	11.7%	11.7%	11.7%	13.3%
DEF	78.6%	82.5%	78.6%	85.4%	14.6%	10.7%	14.6%	8.7%	6.8%	6.8%	6.8%	5.8%

Mesial (*p* = 0.429); buccal (*p* = 0.439); distal (*p* = 0.399); lingual/palatine (*p* = 0.145).

**Table 3:** Modified Plaque Index, 12 months.

	Visible with Probe				Visible				Abundant			
	M	V	D	L/P	M	V	D	L/P	M	V	D	L/P
HEA	74.2%	73.4%	70.3%	81.3%	10.2%	10.2%	10.2%	7.0%	15.6%	16.4%	19.5%	10.9%
DEF	72.8%	78.6%	68.0%	75.7%	12.6%	8.7%	17.5%	11.7%	14.6%	12.6%	14.6%	12.6%

Mesial (*p* = 0.833); buccal (*p* = 0.208); distal (*p* = 0.644); lingual/palatine (*p* = 0.463).

**Table 4:** Modified Bleeding Index, 6 months.

	Punctual				Line in Groove				Profuse			
	M	V	D	L/P	M	V	D	L/P	M	V	D	L/P
HEA	84.4%	82.0%	80.5%	80.5%	13.3%	14.8%	15.6%	15.6%	2.3%	3.1%	3.9%	3.9%
DEF	78.6%	78.6%	75.7%	79.6%	15.5%	14.6%	15.5%	12.6%	5.8%	6.8%	8.7%	7.8%

Mesial (*p* = 0.324); buccal (*p* = 0.307); distal (*p* = 0.427); lingual/palatine (*p* = 0.391).

**Table 5:** Modified Bleeding Index, 12 months.

	Punctual				Line in Groove				Profuse			
	M	V	D	L/P	M	V	D	L/P	M	V	D	L/P
HEA	75.0%	72.7%	75.0%	77.3%	14.8%	19.5%	17.2%	14.8%	10.2%	7.8%	7.8%	7.8%
DEF	77.7%	79.6%	79.6%	81.6%	15.5%	13.6%	17.5%	13.6%	6.8%	6.8%	2.9%	4.9%

Mesial (*p* = 0.665); buccal (*p* = 0.273); distal (*p* = 0.441); lingual/palatine (*p* = 0.619).

**Table 6:** Bone Position/Groups.

	HEA		DEF		*p*-Values
	Mean	SD	Mean	SD	
0 M	−0.7054	0.6253	−0.7969	0.804	0.103
6 M	−0.2358	0.6697	−0.3878	0.6682	
12 M	−0.2169	0.7109	−0.4319	0.7963	

**Table 7:** Bone Position/Abutment Height.

	1 mm		2 mm		*p*-Values
	Mean	SD	Mean	SD	
0 M	−0.6547	0.6338	−0.8434	0.7747	0.02
6 M	−0.1487	0.6072	−0.4679	0.7004	
12 M	−0.2003	0.7892	−0.4322	0.7034	

**Table 8:** Evolution of Implant Stability/Groups.

	HEA		DEF		*p*-Values
	Mean	SD	Mean	SD	
ISQ 0 M	77.18	9309	78.36	8136	0.731
ISQ 6 M	79.67	8188	76.54	9410	
ISQ 12 M	80.07	9019	76.51	12,463	
